# MiL-FISH: Multilabeled Oligonucleotides for Fluorescence *In Situ* Hybridization Improve Visualization of Bacterial Cells

**DOI:** 10.1128/AEM.02776-15

**Published:** 2015-12-22

**Authors:** Mario P. Schimak, Manuel Kleiner, Silke Wetzel, Manuel Liebeke, Nicole Dubilier, Bernhard M. Fuchs

**Affiliations:** a Max Planck Institute for Marine Microbiology, Bremen, Germany; b Energy Bioengineering and Geomicrobiology Research Group, University of Calgary, Calgary, Alberta, Canada

## Abstract

Fluorescence *in situ* hybridization (FISH) has become a vital tool for environmental and medical microbiology and is commonly used for the identification, localization, and isolation of defined microbial taxa. However, fluorescence signal strength is often a limiting factor for targeting all members in a microbial community. Here, we present the application of a multilabeled FISH approach (MiL-FISH) that (i) enables the simultaneous targeting of up to seven microbial groups using combinatorial labeling of a single oligonucleotide probe, (ii) is applicable for the isolation of unfixed environmental microorganisms via fluorescence-activated cell sorting (FACS), and (iii) improves signal and imaging quality of tissue sections in acrylic resin for precise localization of individual microbial cells. We show the ability of MiL-FISH to distinguish between seven microbial groups using a mock community of marine organisms and its applicability for the localization of bacteria associated with animal tissue and their isolation from host tissues using FACS. To further increase the number of potential target organisms, a streamlined combinatorial labeling and spectral imaging-FISH (CLASI-FISH) concept with MiL-FISH probes is presented here. Through the combination of increased probe signal, the possibility of targeting hard-to-detect taxa and isolating these from an environmental sample, the identification and precise localization of microbiota in host tissues, and the simultaneous multilabeling of up to seven microbial groups, we show here that MiL-FISH is a multifaceted alternative to standard monolabeled FISH that can be used for a wide range of biological and medical applications.

## INTRODUCTION

Fluorescence *in situ* hybridization (FISH) is a well-established tool in environmental and medical microbiology (1). Standard FISH involves the hybridization of fluorescently labeled oligonucleotide probes to the rRNA of whole fixed cells, which can subsequently be visualized under an epifluorescence microscope or isolated from a sample by fluorescence-activated cell sorting (FACS) for further analysis, such as single-cell genomics (2–4).

The accurate detection of microbial cells in environmental samples greatly depends on probe signal strength, which correlates with the number of rRNA molecules in a target cell (5). Consequently, many environmental cells with low rRNA copy numbers, such as those of the SAR11 clade, are often close to or below the detection limits (6). Additionally, the identification of bacterial cells associated with plant or animal tissue may be challenging due to high autofluorescence of extracellular matrixes or cellular components, such as cuticle, chitin, or egg yolk.

Catalyzed reporter deposition-FISH (CARD-FISH) was developed to overcome signal limitations via the immobilization of multiple radicalized fluorescent tyramides by the enzyme horseradish peroxidase (HRP) conjugated to oligonucleotide probes (7). The resulting fluorescence signal is 26 to 41 times higher than that with monolabeled FISH, making the visualization of hard-to-detect cells possible (5). However, the use of CARD-FISH to target several groups of interest from a single sample is very time-consuming, as consecutive hybridizations for each target group need to be made.

Standard FISH approaches have also undergone development to increase signal through a variety of probe synthesis advancements. A previous study conjugated a 3-times-labeled fluorescence tail at one end of the probe (8). The use of these probes, however, considerably increased nonspecific binding, presumably due to dye moieties binding to cell components, and it was therefore not practical for environmental samples. Another approach, called double labeling of oligonucleotide probes (DOPE)-FISH, uses probes labeled at the 5′ and 3′ ends to increase signal by 2-fold and is not compromised by nonspecific binding of dye moieties (9). In addition to signal increase, probes for DOPE-FISH can be multilabeled, allowing multicolor probe combinations. Behnam and coworkers (10) showed that DOPE oligonucleotides can be used to target up to six organisms in a single FISH experiment by conjugating various combinations of the standard fluorescein, Cy3, and Cy5 fluorochromes to a probe. Multicolor labeling is also commonly used in human cytogenetics and can be achieved in a number of ways: (i) a combinatorial approach, whereby the number of available fluorochrome labels determines the number of targets (2^*n*^ − 1) (11); (ii) ratio labeling, whereby the proportion of each dye used determines a ratio code, which is allocated to a target molecule (12); or (iii) a combination of both combinatorial and ratio labeling, such as combined binary ratio labeling (COBRA-FISH) (13, 14). To further increase the number of environmental organisms one can target in microbial ecology studies, combinatorial labeling and spectral imaging FISH (CLASI-FISH) can be employed (15). With CLASI-FISH, a target group is allocated a unique spectral tag by hybridizing a repertoire of monolabeled oligonucleotide probes carrying fluorochromes of closely overlapping spectra to several sites on the 16S rRNA. The combination of emitted wavelengths is revealed by linear unmixing and spectral imaging with modern confocal laser scanning microscopes (CLSM). However, approaches that rely on the targeting of several sites on a target molecule, such as the 16S rRNA, are generally negatively influenced by binding sensitivity and specificity biases.

The preservation of microbial community composition in, for example, biofilms or within eukaryotic tissue is critical for understanding these systems as a whole. In particular, FISH-compatible acrylic resins, such as LR white, preserve specimen structure and enable semithin sectioning (1 μm) without risking cell displacement, which allows the identification and localization of individual microbial cells within a community (16–18). In animals and plants, however, the high autofluorescence of some tissues, extracellular matrixes, or cellular components, such as muscle, cuticle, chitin, or egg yolk, can quench probe signal, making the visualization of microbial cells challenging. The development of a FISH approach that simultaneously and reliably targets multiple bacterial phylotypes, yields strong probe signals over high background autofluorescence, and optimally preserves cell morphology on specimens embedded in acrylic resins is therefore highly desirable for studies of beneficial and pathogenic microbiota associated with plants and animals.

In this study, we demonstrate the application of multilabeled oligonucleotide probes synthesized by copper-catalyzed alkyne azide 1,3-dipolar cyclo-addition (CuAAC) or “click” reaction (19) on complex microbial populations and termed this procedure multilabeled FISH (MiL-FISH). We investigated signals and the melting behavior of probes labeled with up to four fluorochromes and show that signal strength was sufficient for the isolation of unfixed environmental microbes via FACS. For optimal localization of microorganisms in their natural environment, differences between mono-, MiL-, and CARD-FISH were evaluated on bacteria associated with animal tissue embedded in FISH-compatible acrylic resin. For these analyses, we used the well-studied symbiosis in the gutless oligochaete Olavius algarvensis (20). This marine worm is nutritionally dependent on a consortium of two gammaproteobacterial and two deltaproteobacterial symbionts (21). Attempts at tracing the transmission mode and the association of symbionts with the developing worm embryo using monolabeled FISH have been challenging due to the high autofluorescence of egg yolk. Finally, to demonstrate the ability of MiL-FISH to target up to seven members of a microbial community, we hybridized a mock consortium of marine microorganisms with probes carrying a variety of combinations of the most commonly used fluorochromes, fluorescein (6-FAM), Cy3, and Cy5.

## MATERIALS AND METHODS

### Sample material.

Seven bacteria, Escherichia coli strain DSM 498, Gramella forsetii strain KT0803, Beggiatoa sp., Desulfococcus biacutus, Roseobacter sp. strain AK199, Sulfurimonas denitrificans, and Rhodopirellula sp. strain SH1^T^, and one archaeon, Metallosphaera sedula, were used in this study (for culture preparation and fixation, see the supplemental material).

For the isolation of environmental microorganisms by FACS, we used the symbiotic bacteria of the gutless marine worm Inanidrilus leukodermatus (22). Roughly 1,000 worms were sampled in November 2009 from shallow-water sediments (1-m water depth) in Harrington Sound on Bermuda, next to the Bermuda Aquarium (32°19′26.52″N, 64°44′18.78″W). Worms were homogenized and symbionts partially separated from host tissue via Histodenz density gradient centrifugation, according to the protocol described by Kleiner et al. (23). Symbiont fraction pellets were kept frozen at −80°C until further analysis.

The gutless marine oligochaete O. algarvensis was retrieved from sediments collected at 7-m water depth by scuba diving in the bay of Sant'Andrea, Elba, Italy (42°48′29.80″N, 10°8′34.07″E). The worms were sorted from the sediment by hand at the HYDRA field station (Fetovaia, Elba, Italy) and fixed in Carnoy's fixative (6 parts 96% ethanol, 3 parts chloroform, 1 part glacial acetic acid) and 4% paraformaldehyde (PFA) for 4 h each at 4°C and stored in 50% ethanol-seawater or preserved directly in 96% ethanol. Eggs were obtained by incubating worms in sediment-filled 78-μm-pore-size vials (Plano GmbH, Wetzlar, Germany), which were subsequently buried in the sediment of an aquarium of 39‰ salinity kept at 25°C with a cooling aggregate (Titan 150; Aqua Medic GmbH, Bissendorf, Germany). Vials were sampled every week and manually screened for eggs. Eggs at different developmental stages were retrieved and fixed as described above.

### Oligonucleotide probes.

Sequences for the previously published general probes used in this study were obtained from probeBase (24) (see Table S1 in the supplemental material). All probes in this study, including multilabeled probes, were donated by or purchased from biomers.net GmbH (Ulm, Germany) and synthesized with up to four click-compatible nucleotides (A, T, C, and/or G). Multicolored probes were synthesized with two internal click-compatible nucleotides and two fluorochromes of a different species on the 5′ and 3′ ends (DOPE) (see Table S1 for positions and dyes used). Probes subsequently underwent a “click” reaction with the respective dye and were purified before use.

### LR white embedding.

O. algarvensis worms and eggs were embedded by gradual ethanol dehydration starting at 50% for 10 min, followed by 10% increases for 10 min each, and the final dehydration step at 100% ethanol was repeated three times. Next, infiltration of resin into dehydrated samples was performed on a shaker with an LR white-ethanol mix of 1:3, 1:2, 1:1, 2:1, and 3:1 for 30 min each, followed by pure LR white four times for 1 h each, with the last change left overnight. Samples were orientated in gelatin capsules with fresh LR white, placed into a desiccator, and flushed with argon three times, and a 1 × 10^5^-Pa vacuum was applied before polymerization at 50°C in an oven for 3 days. Semithin (1-μm) sectioning of embedded specimens was done on an ultramicrotome (UC7; Leica Microsystems, Vienna, Austria). The sections were placed on a drop of 20% acetone on gelatin-chrome alum [KCr(SO_4_)_2_·12H_2_O]-coated glass slides and dried on a hot plate at 50°C.

### FISH.

Hybridization of cells with multilabeled oligonucleotide probes was performed using a slight alteration of the standard FISH protocol, as described by Manz et al. (25). Hybridization buffer (900 mM NaCl, 20 mM Tris-HCl [pH 7.5], 0.02% sodium dodecyl sulfate [SDS]) with a formamide concentration optimized for individual probes used in each respective experiment (see Table S1 in the supplemental material) was mixed with 8.4 pmol μl^−1^ oligonucleotide working solution at a ratio of 15:1. The effect of CARD-FISH buffer on multilabeled probes was tested by adding 10% (wt/vol) dextran sulfate and 1% (wt/vol) blocking reagent (Boehringer, Mannheim, Germany). All hybridizations were conducted at 46°C in a humid chamber for 3 h (unless otherwise stated), followed by a 10-min wash at 48°C with washing buffer, according to Manz et al. (25) (14 to 900 mM NaCl, 20 mM Tris-HCl [pH 8], 5 mM EDTA [pH 8], and 0.01% SDS), adjusted to the stringency of the formamide concentration used. Hybridization of resuspended I. leukodermatus symbiont pellets was done as described above, with the addition that cells were pelleted for each buffer exchange for 15 min by centrifugation at 1,500 × *g*. This step was necessary because cells for FACS were required to be in aqueous solution and therefore could not be immobilized on filters during the FISH procedure.

CARD-FISH experiments were conducted based on the protocol described in Pernthaler et al. (7), with slight modifications. Hybridization buffer (900 mM NaCl, 20 mM Tris-HCl [pH 7.5], 0.02% sodium dodecyl sulfate [SDS], 10% [wt/vol] dextran sulfate, and 1% [wt/vol] blocking reagent) with 8.4 pmol μl^−1^ probe at a ratio of 150:1 was applied. Hybridization at 46°C for 3 h and a 10-min wash step at 48°C with the adjusted wash buffer (14 to 900 mM NaCl, 20 mM Tris-HCl [pH 8], 5 mM EDTA [pH 8], and 0.01% SDS) followed. Amplification buffer (1× phosphate-buffered saline [PBS; pH 7.3], 0.0015% [vol/vol] H_2_O_2_, 1% Alexa Fluor 488 or 594 dye [Thermo Fisher Scientific, Waltham, MA, USA]) was prepared and cells were incubated for 1 h at 46°C in a humid chamber until a final wash for 10 min in 1× PBS.

For mono-, MiL-, and CARD-FISH performed on LR white-embedded O. algarvensis specimens, a PAP pen (Science Services, Munich, Germany) was used to draw rings around 1-μm sections mounted on glass slides. The sections were pretreated in 200 mM HCl for 10 min, washed in 200 mM Tris-HCl for 10 min, incubated in 1 μg/ml proteinase K for 5 min at 46°C, and finally washed again in 200 mM Tris-HCl for 10 min before the protocols described above were applied.

### Microscopy.

Fluorescence images were taken with an AxioCam Mrm camera mounted on an Axioscope2 epifluorescence microscope (Carl Zeiss AG, Oberkochen, Germany) equipped with Fluos Zeiss 09 (excitation [ex], 470/40 nm; emission [em], 515LP), AHF (AHF analysentechnik AK, Tübingen, Germany), F36-525 Alexa 488 (ex, 472/30 nm; em, 520/35 nm), AHF F46-004 Cy3 (ex, 545/25 nm; em, 605/70 nm), and AHF F46006 Cy5 (ex, 620/60 nm; em, 700/75 nm) filter sets. Images were recorded with the PC-based AxioVision (release 4.6.3 SP1) imaging software and any image-level adjustments made therein.

### FACS.

Unfixed hybridized microbial cells of I. leukodermatus were sorted into a 1.5-ml Eppendorf tube using a MoFlo flow cytometer (DakoCytomation, Hamburg, Germany). The laser was set to λ 488 nm at 400 mW and used with a 488/6 filter cube for side scatter (SSC) and 531/40 for Atto488 fluorescence in dual trigger mode.

### Label-dependent intensity.

To assess probe-conferred signals of 1-, 2-, 3-, and 4-times-labeled EUB338 oligonucleotide probes (MiL-FISH probes), three reference strains, E. coli DSM498, G. forsetii KT0803, and Roseobacter sp. AK199, were each hybridized in triplicate. The fluorochrome positions on the probes are listed in Table S1 in the supplemental material. Cells were resuspended in 10 ml of 1× PBS and filtered onto 42-mm GTTP filters with a 0.22-μm pore size (Millipore, Darmstadt, Germany). Filters were cut into eighths and placed on a piece of Parafilm (Brand, Wertheim, Germany) on a glass slide. FISH was conducted on each filter piece, and 56 μl of hybridization buffer and 4 μl of probe were added. After washing, the filter pieces were placed onto new glass slides with a mounting medium mix consisting of 11 parts CitiFluor (CitiFluor Ltd., London, England), two parts VectaShield (Vector Laboratories, Burlingame, CA, USA), and one part 1× PBS (pH 9) to prevent bleaching of pH-sensitive dyes, and these were sealed with a coverslip before microscope analysis. To avoid oversaturation of signal by strong fluorescence of the multilabeled probes, images were recorded at exposure times that were optimal for each label type and later correlated to each other. The recorded images were evaluated with ACMEtool2 build 1.0.0.42 (http://www.technobiology.ch/index.php?id=acmetool).

### Melting curves.

The melting behaviors of multilabeled probes were determined in triplicate with 1-, 2-, and 4-times-fluorescein-labeled CF319a probe targeting G. forsetii. The filters were prepared as described above, with hybridization conditions ranging from 20 to 70% formamide in 10% step increases. Epifluorescence microscopy followed, and images were analyzed with the daime 2.0 software package (26).

### MiL-FISH for multicolor detection of seven microbial groups.

For the detection of multiple microbial cells, probes were labeled with fluorescein, Cy3, and Cy5 only and combinations of fluorescein and Cy3; fluorescein and Cy5; Cy3 and Cy5; and Cy3, Cy5, and fluorescein. The recording of signals in the respective channels and overlaying all channels of an image result in the visualization of mixed colors. For example, a probe labeled with fluorescein and Cy3 yields a yellow overlay signal in positively hybridized cells, whereas a probe labeled with Cy3 and Cy5 produces magenta when Cy5 is set to blue. The strains, probes, and fluorochrome combinations used are listed in Table S1 and shown in Fig. S3 in the supplemental material. For optimal stringency, two sequential hybridizations were performed, the first at 60% formamide for DSS658 and the second at 35% formamide for the remaining six probes.

## RESULTS

### Label-dependent intensity of MiL-FISH.

**FIG 1:**
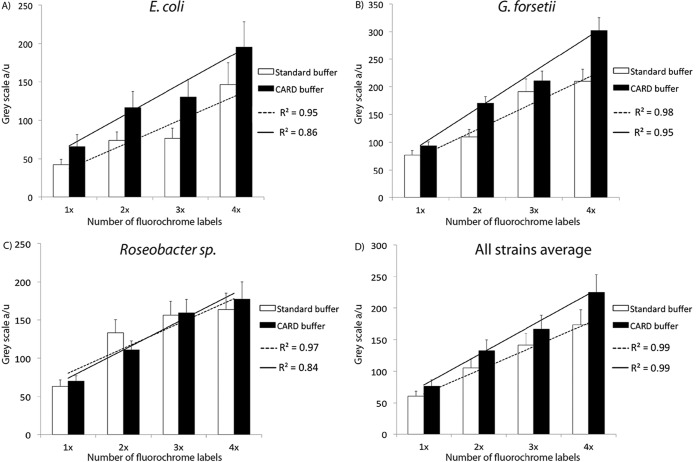
Signal intensities of MiL-FISH probes with 1 to 4 fluorescent labels. Three reference strains, E. coli DSM498 (A), G. forsetii KT0803 (B), and Roseobacter sp. AK199 (C), were hybridized in triplicate with a 1-, 2-, 3-, and 4-times-fluorescein-labeled EUB338 MiL probe using both standard (white bars) and CARD-FISH (black bars) hybridization buffer. (D) Average signal increase in all three reference strains. The solid line shows the linear regression of CARD-FISH buffer on cells, and the dashed line shows the linear regression of standard FISH buffer. Error bars indicate standard deviations for triplicate hybridizations. a/u, arbitrary units.

The 2-, 3-, and 4-times-labeled EUB338 probe yielded on average 1.8-, 2.3-, and 2.9-fold signal increases, respectively, over that of a 1-time-labeled probe with standard FISH hybridization buffer (Fig. 1). The G. forsetii hybridization yielded the strongest signal, followed by Roseobacter sp. AK199 and then E. coli. For all labels and organisms, an average linear increase proportional to the number of labels was measured (*R*^2^ = 0.99). The use of CARD-FISH buffer during hybridization yielded a 20% increase in signal for all labels and reference strains (Fig. 1). This indicates that CARD-FISH buffer increases signal by the same factor, independent of the number of labels added to a probe.

For all experiments, a 4-times-labeled NON-EUB negative control that yielded no visible signals in cells was included. In our experiments, the signal-to-noise (S/N) ratios between the negative controls and G. forsetii cells hybridized with the CF319a probe carrying various label numbers were as follows: 1-time-labeled probe, 4.3; 2-times-labeled probe, 13.2; 3-times-labeled probe, 23.6; and 4-times-labeled probe, 34.7 (see Table S2 in the supplemental material).

**FIG 2:**
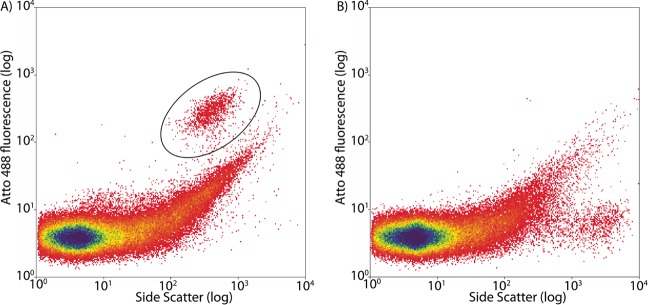
(A) FACS plot of 4-times-Atto488-labeled EUB338 probe targeting symbionts of I. leukodermatus. The circled region shows the fraction of labeled bacterial cells. (B) Corresponding negative control with 4-times-Atto488-labeled NON338 probe.

To demonstrate the efficiency of multilabeled probes for fluorescence-activated cell sorting in a flow cytometer, microbial symbionts of the gutless oligochaete I. leukodermatus were hybridized unfixed directly after worm homogenization. Separation from host tissue was evident by a clear cluster of labeled microbial cells above background fluorescence that were distinct from negative controls hybridized with a 4-times-Atto488-labeled NON338 probe (Fig. 2). Visual inspection under an epifluorescence microscope confirmed that the sorted cells were free of host tissues, cells, and other visible host components.

### Melting behavior of MiL-FISH probes.

Melting curves for the 1-, 2-, and 4-times-fluorescein-labeled probe CF319a were generated with G. forsetii cells and standard hybridization buffer. All fluorescein-labeled probes showed a characteristic decrease of signal with between 30% and 40% formamide, as previously described for CF319a with mono-FISH (27) (see Fig. S1 in the supplemental material).

### LR white FISH.

Differences between FISH with mono-, multiple-, and HRP-labeled probes of resin-embedded material were evaluated by hybridizing gammaproteobacterial sulfur-oxidizing and deltaproteobacterial sulfate-reducing microbial symbionts of O. algarvensis.

**FIG 3:**
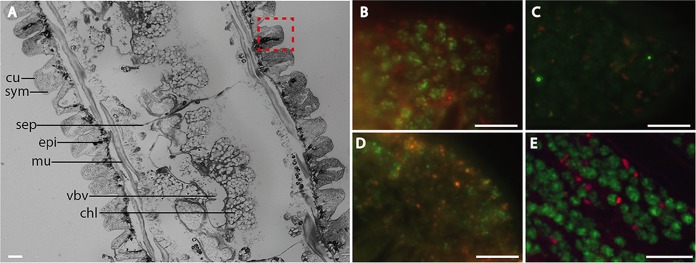
Comparison of mono-, MiL-, and CARD-FISH performed on LR white-embedded O. algarvensis cross sections. (A) Ethanol-preserved specimen. cu, cuticle; sym, symbionts; sep, septum; epi, epidermis; mu, muscle; vbv, ventral blood vessel and nerve chord; chl, chloragogen tissue. The dashed red square defines an example of the regions shown in panels B to E. (B to E) Gam42a (green) and DSS658 (red) oligonucleotide probes used on ethanol-preserved specimens with a hybridization time of 19 h (B) or 3 h (C), or with CARD-FISH (D) and MiL-FISH (E) specimens fixed in Carnoy’s fixative and PFA and hybridized for 3 h. Scale bars, 5 μm.

For standard FISH with monolabeled probes, two hybridization times were tested on LR white-embedded worms fixed with Carnoy's fixative-PFA and stored in 50% ethanol and with worms conserved directly in 96% ethanol. None of the PFA-fixed individuals showed positive signals with monolabeled oligonucleotides. In contrast, a 19-h hybridization of a 96% ethanol-preserved specimen based on the report by Schimak et al. (17) yielded clearly visible signals of bacterial cells by use of the standard FISH protocol with CARD-FISH buffer (Fig. 3B). A 3-h hybridization of ethanol-preserved material yielded signal for the DSS658 probe but none for the Gam42a probe (Fig. 3C). For both hybridization times, long exposure during image acquisition was required, increasing the signal-to-noise ratio and dampening overall probe signals.

CARD-FISH on LR white sections yielded strong signals at a hybridization time of 3 h on PFA-fixed worms (Fig. 3D). However, microbial cell morphology was compromised by a “patchy” distribution of probe signal, making the identification of individual cells within the bacterial consortia difficult.

MiL-FISH with a hybridization time of 3 h on PFA-fixed worms overcame problems with image quality and cell integrity (Fig. 3E). Single microbial cells were clearly visible within the consortia and could easily be differentiated from each other. Reduced exposure times compared to those with monolabeled probes (<25%) were sufficient during imaging, increasing the S/N ratio by 53% over that of mono-FISH.

**FIG 4:**
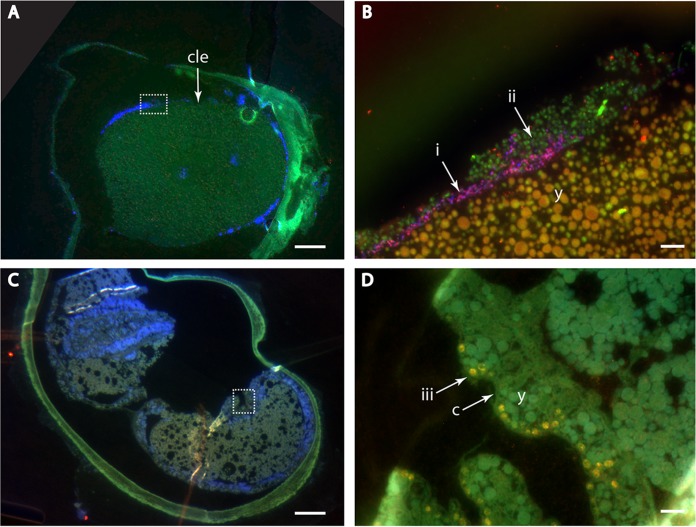
One-micrometer sections of LR white-embedded O. algarvensis eggs. (A) DAPI (4′,6-diamidino-2-phenylindole)-stained overview of egg after first cleavage. The dashed white rectangle defines the region shown in panel B. cle, cleavage. (B) Gammaproteobacteria (ii) and deltaproteobacteria (i) hybridized with 4-times-labeled Gam42a and 4-times-labeled DSS658 probe, respectively. The autofluorescence of the egg yolk is overcome, and bacteria are seen to associate closely with the developing embryo. y, egg yolk. (C) DAPI-stained overview of juvenile worm in egg. The dashed white rectangle defines the region shown in panel D. (D) Gamma 1 symbiont phylotype (iii) hybridized with 16S rRNA-specific probe labeled 2 times with fluorescein and 2 times with Cy3 to produce yellow in the overlay. Symbiont cells are incorporated between the cuticle and epidermis and in close proximity to the egg yolk. y, egg yolk; c, cuticle. Scale bars, 50 μm (A and C) and 5 μm (B and D).

Further, probe signals were high enough to overcome tissue autofluorescence, enabling the accurate localization and identification of individual microbial cells. MiL-FISH on eggs embedded in LR white resulted in clear signals of gamma- and deltaproteobacteria around high-autofluorescence regions, such as egg yolk (Fig. 4B). Gamma 1 symbiont phylotypes in close proximity to both egg yolk and cuticle were clearly visible in a juvenile worm by use of a specific 16S rRNA probe double labeled with 2× fluorescein and 2× Cy3, producing a yellow overlay (Fig. 4D; see also Table S1 in the supplemental material).

### MiL-FISH for multicolor detection of seven microbial groups.

**FIG 5:**
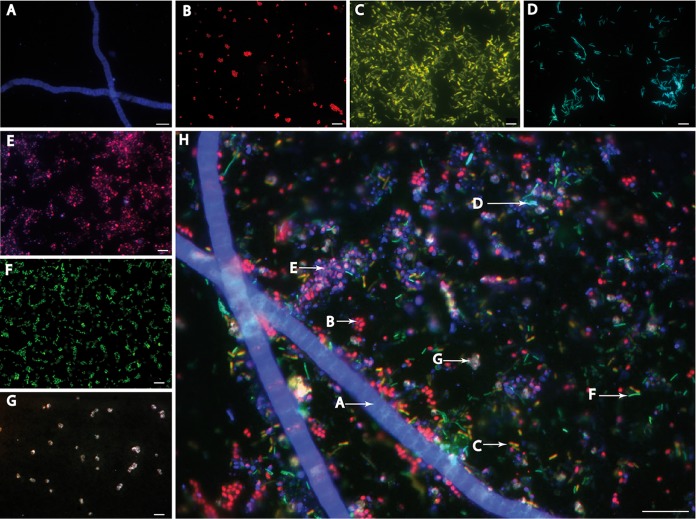
Epifluorescence images of MiL-FISH–labeled microorganisms as listed in Table S1 in the supplemental material and shown here. Shown are hybridizations of Beggiatoa sp. with Gam42a (A), D. biacutus with DSS658 (B), Roseobacter sp. with Ros537 (C), S. denitrificans with EPSY914 (D), Rhodopirellula sp. SH1^T^ with PLA46 (E), G. forsetii with CF319a (F), and M. sedula with Arch915 (G). (H) Composite image of all seven microbial partners in an artificial mix. The letters correlate with images of individual organisms shown in panels A to G. Scale bars, 10 μm (A and H) and 5 μm (B to G).

A mock mixture of seven marine microbial groups of different sizes and morphologies was hybridized with MiL-FISH probes labeled with different fluorochrome combinations. Our experiments showed that each member of the mixture could be distinguished based on the specific color spectrum emitted by the combination of fluorochromes allocated to the probes, as shown in Fig. 5 (see also Fig. S3 and Table S1 in the supplemental material). A comparison of the cell morphology and signal of the respective probe was used to confirm positive hybridization of the bacterial cells. Further, the visual control for positive hybridization was compared with the other six organisms in the mixture that acted as negative controls. The exposure times for each channel in the final overlay image were set to accommodate all wavelengths and signal intensities for fluorochromes used in the experiment, as follows: fluorescein, 213 ms; Atto488, 1,197 ms; Cy5, 435 ms; and Cy3, 123 ms.

The hybridization of G. forsetii with a 4-times-fluorescein-labeled CF319a probe yielded optimal signals at an exposure time of ∼100 ms. This was, however, not sufficient to visualize the 2-times-fluorescein-labeled component of mixed probes containing two fluorochrome species, such as Ros537, causing incorrect color representation of these cells in the final overlay image. Therefore, a 2×-fluorescein-labeled CF319a probe was used in the final probe mix, and a ∼200-ms exposure time was applied (Fig. 5H; see also Table S1 in the supplemental material). This observation was not made for probes labeled with a Cy3 dye. Adding a 4-times-Cy3-labeled DSS658 probe that targeted D. biacutus to the probe mix did not negatively influence the 2-times- Cy3-labeled component of the mixed probes.

## DISCUSSION

In our study, a maximum of four fluorochromes per probe was tested, yielding an average 2.9-fold signal increase over a 1-time-labeled probe. Standard FISH on most environmental cells yields a signal-to-noise ratio (S/N ratio) of around 4. In our experiment, the S/N ratio was improved by a factor of 8.5, and one-quarter-shorter exposure times were required to visualize cells with a 4-times-labeled probe. Photochemical quenching may have influenced the overall signal yield; however, we assume that quenching was minimized by placing dye moieties along the entire length of the probe. Theoretically, probes can be labeled with at least twice the number of labels. However, the point at which additional labeling yields no further signal increase is still open to investigation. The results from our negative controls indicate that dye moieties did not adhere to cellular components, as shown in past studies (8), making nonspecific binding a negligible factor for MiL-FISH probes. To further increase the probe signal hybridization buffer that contains dextran sulfate (like for CARD-FISH), a known crowding reagent that reduces the time required for probe annealing (28) can be used. Unlike HRP-labeled probes that are used for CARD-FISH, multilabeled probes did not significantly affect melting behavior (see Fig. S1 in the supplemental material). Specific testing of additional probes and organisms may be advisable, but based on our experience, we expect no differences in optimal hybridization conditions for already-published probes from sources, such as probeBase (29).

In mono-FISH, a minimum of ∼400 probe-rRNA hybrids are necessary for a detectable signal of cells using modern epifluorescence microscopes (5). This implies that with a 4-times-labeled MiL-FISH probe, a minimum of only ∼130 probe-rRNA hybrids results in the same signal yield. Thus, MiL-FISH probes have the potential to increase the detection of organisms with low ribosomal content in environmental samples, such as members of the SAR11 clade (30). Additionally, unfixed environmental bacteria hybridized with MiL-FISH probes were detectable and isolated by flow cytometric sorting. Flow cytometers have lower sensitivity than that of epifluorescence microscopes and require stronger probe signals for the positive detection of hybridized cells.

MiL-FISH on LR white sections yielded stronger signals than those of monolabeled probes and clearer bacterial cell morphology than that of CARD-FISH for symbionts of adult O. algarvensis worms. CARD-FISH signals were strong, but individual microbial cells could not be clearly distinguished from each other. As there is no probe penetration into LR white sections (31), access to cellular components requires chemical etching, usually with hydrochloric acid, to expose target rRNA on the section surface, onto which probes can bind. The amplification process in CARD-FISH radicalizes tyramides, which bind to intracellular proteins. We speculate that target molecules for tyramides are not homogenously accessible after etching, resulting in the observed patchy signal and suboptimal visualization of bacterial cell morphology. Analysis of O. algarvensis eggs revealed that both gamma- and deltaproteobacterial symbionts occur on the blastomeres of the developing embryo from as early as the first cleavage. This might indicate that they play an important role in the developmental processes of their host, as shown for other symbiotic associations (i.e., 32). The distribution of individual symbiont cells was clearly visible, and a distinction between bacterial phylotypes within the consortia was easily made (Fig. 4B). The MiL-FISH combinatorial labeling concept identified the primary gammaproteobacterial symbiont (Gamma 1) in the juvenile host by use of a probe labeled with fluorescein and Cy3. As with the multicolor detection of seven bacteria in our mock mix, we are now able to simultaneously identify and visualize all four symbiont phylotypes and overcome limitations, such as background autofluorescence. These findings pave the way for a more detailed analysis of the roles that individual microbes play in the establishment of this symbiosis. Our results show that MiL-FISH can be used in combination with acrylic resins to identify and localize host-associated bacteria, making this approach useful for many biological and medical applications.

A past study (33) targeted seven closely related betaproteobacteria, with three probes carrying individual label types targeting three sites on the 16S rRNA. However, difficulties may arise in the probe design of highly conserved 16S rRNA regions of closely related species and the targeting of phylogenetically distant groups. MiL-FISH enabled the visualization of seven microbial groups by combinatorial labeling of probes and eliminated sensitivity biases, as only one site on the 16S rRNA was targeted. Probe signal strength depends on the rRNA content of a target cell, and differences between organisms must be considered in multicolor hybridization of a large bacterial community. To account for rRNA variation, we suggest first hybridizing with individual probes and carefully monitoring exposure times before a multicolored MiL-FISH approach is applied to environmental samples. Large differences between cell signals can subsequently be adjusted by reduction or addition of the corresponding fluorochrome label on probes used for multicolor imaging.

CLASI-FISH can be employed to target even more than seven species in environmental samples, and it relies heavily on an even accessibility of probes to multiple sites on the 16S rRNA. The variability in binding efficiency and accessibility to different 16S rRNA target sites (34, 35) can result in false-negative binding of probes and/or false and incomplete spectral allocation to a target group. A solution has been described to overcome this by using a mix of monolabeled probes carrying a variety of label types. However, because in this case, only a single site is targeted, probe-probe competition and a linear reduction in fluorescence correlating to the number of dyes added are expected (36). The use of MiL-FISH probes in CLASI-FISH would greatly streamline this concept and reduce the time needed for probe design and testing of multiple target sites on the 16S rRNA while offering high probe signals. Several strategies can be employed in combining MiL- and CLASI-FISH, as follows. (i) A single site is targeted by a 4-times-labeled probe, resulting in the same spectral identity as four monolabeled probes targeting four sites on the 16S rRNA. This greatly increases specificity and sensitivity, as only one target site is used, and by placing dye moieties along the length of the probe, several dozen label combinations can be utilized (see Fig. S2A1 and A2 in the supplemental material). Indeed, this concept can be expanded to ratio labeling or combined combinatorial and ratio labeling approaches, such as COBRA-FISH (13), which might also greatly benefit from the use of MiL-FISH probes by allocating ratio combinations to the probe itself. (ii) If several sites on the 16S rRNA are available, a signal increase can be achieved by the addition of 4-times-labeled probes carrying the same fluorochrome combination for each target site. This potentially allows the inclusion of environmental cells with low ribosomal content in future CLASI-FISH studies (see Fig. S2B in the supplemental material). (iii) The targeting of multiple 16S rRNA sites using MiL-FISH probes with a large variety of fluorochrome combinations could increase available spectral identities by 4-fold (see Fig. S2C in the supplemental material).

## Supplementary Material

Supplemental material
