# Supplementary material for: MiL-FISH: Multilabeled Oligonucleotides for Fluorescence In Situ Hybridization Improve Visualization of Bacterial Cells
Source: Appl Environ Microbiol. 2015 Dec 22;82(1):62–70. doi: 10.1128/AEM.02776-15 (PMC4702640; doi:10.1128/AEM.02776-15)
Supplement: Supplemental material [file supp_82_1_62__index.html]

MiL-FISH: Multilabeled Oligonucleotides for Fluorescence In Situ Hybridization Improve Visualization of Bacterial Cells — Supplemental material 

# MiL-FISH: Multilabeled Oligonucleotides for Fluorescence *In Situ* Hybridization Improve Visualization of Bacterial Cells

## Supplemental material

- Supplemental file 1 -

  Supplemental materials and methods, oligonucleotide probes (Table S1), signal-to-noise ratios (Table S2), melting curve (Fig. S1), three optimization approaches for CLASI-FISH using MiL-FISH probes (Fig. S2), and schematic of multilabeled oligonucleotide probes and target organisms for hybridization of seven marine microbial groups (Fig. S3).

  PDF, 6.5M
