# Supplementary material for: MiL-FISH: Multilabeled Oligonucleotides for Fluorescence In Situ Hybridization Improve Visualization of Bacterial Cells
Source: Appl Environ Microbiol. 2015 Dec 22;82(1):62–70. doi: 10.1128/AEM.02776-15 (PMC4702640; doi:10.1128/AEM.02776-15)
Supplement: Supplemental material [file AEM.02776-15_zam999116797so1.pdf]

Supplementary Information:

Bacterial cultures and sample material:

The gammagroteobacteria, *Escherichia coli* DSM498, was grown in Luria-Bertani medium for 4h 20 at 37°C. At the end-log phase and an optical density of 0.468 (600nm) cells were harvested at 1500g for 10 min., fixed in 2% formaldehyde (v/v) for 1h at RT, washed three times in 1xPBS and pelleted at 1500g for 5 min. Fixed cells were re-suspended in 1xPBS / ethanol (50%) and stored at -20°C until further analysis.

The flavobacteria, *Gramella forsetii* KT0803, was grown on SY-medium containing 40g/L sea salt (Sigma-Aldrich, St. Louis, MO, USA) and 0.1% yeast extract. To avoid cell clumping and after growth at RT for 60h to an optical density of 0.146 (600 nm), 1ml was used as an inoculant in 100ml SY-medium and re-grown to an optical density of 0,065 (600nm). Cells were harvested by centrifugation at 1500 g for 10 min, fixed in 2% formaldehyde for 1h at RT, washed 3 times in 1x PBS and pelleted at 1500g for 10 min. Cells were re-suspended in 50% 1xPBS / ethanol (50%) and stored at -20°C until further analysis.

The planctomycetes, *Rhodopirellula baltica* $SH1^T$, was grown in medium M9 as previously described by Schlesner et al. 1986 **(1)**. Cells were harvested at 1500g for 10 min, fixed in 2% formaldehyde (v/v) for 1h at RT, washed three times in 1xPBS and pelleted at 1500g for 5 min. Fixed cells were re-suspended in 1xPBS / ethanol (50%) and stored at -20°C until further analysis.

The alphaproteobacteria, *Roseobacter sp.* AK 199, plate cultures were picked and grown in 300 ml Difco™ Marine Broth 2216 (Beckton, Dickinson and Company,

New Jersey, USA) for 5 days on a shaker at 28 °C. 10ml of the culture stock was taken and centrifuged at 4000g for 10 minutes and re-suspended in 800 µl 1x PBS. Cells were fixed in 4% PFA for 1 hour at room temperature washed three times in 1x PBS and re-suspended in 50% EtOH / PBS.

The deltaproteobacteria, *Desulfococcus biacutus* DSM5651, were cultured after the guidelines for anaerobic cultivation at 30°C as described on www.dsmz.de for the given reference number DSM5651. Cells were harvested at 1500g for 10 min., fixed in 2% formaldehyde (v/v) for 1h at RT, washed three times in 1xPBS and pelleted at 1500g for 5 min. Fixed cells were re-suspended in 1xPBS / ethanol (50%) and stored at -20°C until further analysis.

The gammaproteobacteria, *Beggiatoa sp.*, were grown with 4 mMol H_2S final concentration after Schwedt et al. 2012 **(2)**, in the presence of *Pseudovibrio sp.* Cells were hand picked, washed three times in 1x PBS and transferred onto 10 well Diagnostika (Thermo Fisher Scientific, MA, USA) gelatine / chromalaune ($KCr(SO_4)_2 \cdot 12H_2O$) coated glass slides. Fixation followed with 4% PFA at room temperature for 1 hour and washed three times in 1 x PBS.

The epsilonproteobacteria, *Sulfurimonas denitrificans*, was grown as described for DSMZ strain DSM1251 (http://www.dsmz.de/catalogues/details/culture/DSM-1251.html). 10ml of the culture stock was taken and centrifuged at 4000g for 10 minutes and re-suspended in 800 µl 1x PBS. Cells were fixed in 4% PFA for 1 hour at room temperature washed three times in 1x PBS and re-suspended in 50% EtOH / PBS.

The sulfolobales (Archaea), *Metallosphaera sedula* DSM 5348^T, was grown as described by Huber *et al.* 1989 **(3)** without sulfur particles in the media as previously described by Behrens *et al.* 2003 **(4)**. Cells were harvested at 1500g for 10 min., fixed in 2% formaldehyde (v/v) for 1h at RT, washed three times in 1xPBS and pelleted at 1500g for 5 min. Fixed cells were re-suspended in 1xPBS / ethanol (50%) and stored at -20°C until further analysis.

Probe	Sequence 5' - 3' (reverse complementary)	Target gene	Label	Synthesis	Taxon	Target Species	FA %	Colour
DSS658	TCC ACT TCC CTC TCC CAT	16S rRNA	4x Cy3	Click chemistry	Delta-proteobacteria	*Desulfococcus biacutus*	60%	Red
Gam42a	GCC TTC CCA CAT CGT TT	23S rRNA	4x Cy5	Click chemistry	Gamma-proteobacteria	*Beggiatoa sp.*	35%	Blue
CF319a	TGG TCC GTG TCT CAG TAC	16S rRNA	4x 6-FAM	Click chemistry	Flavobacteria	*Gramella forsetii*	35%	Green
CF319a	TGG TCC GTG TCT CAG TAC	16S rRNA	5' & 3' 6-FAM	DOPE	Flavobacteria	*Gramella forsetii*	35%	Green
CF319a	TGG TCC GTG TCT CAG TAC	16S rRNA	5' 6-FAM	mono	Flavobacteria	*Gramella forsetii*	35%	Green
Ros537	CAA CGC TAA CCC CCT CC	16S rRNA	5' & 3' end 6-FAM, 2x Cy3 intern	DOPE & Click chemistry	Alpha-proteobacteria	*Roseobacter sp.* AK 199	35%	Yellow
EPSY914	GGT CCC CGT CTA TTC CTT	16S rRNA	5' & 3' end 6-FAM, 2x Cy5 intern	DOPE & Click chemistry	Epsilon-proteobacteria	*Sulfurimonas denitrificans*	35%	Cyan
PLA46	GAC TTG CAT GCC TAA TCC	16S rRNA	5' & 3' Cy3, Cy5 intern	DOPE & Click chemistry	Planctomycetes	*Rhodopirellula sp.* SH^{1} T	35%	Magenta

Arch915	GTG CTC CCC CGC CAA TTC CT	16S rRNA	2x Cy5 intern, 5' Cy3, 3' Atto 488	DOPE & Click chemistry	Archaea	*Metallosphaera sedula*	35%	White
Oalg_G1_644_20mer	TAC CAC ACT CTA GCC GGA CA	16S rRNA	5' & 3' 6-FAM, Cy3 intern	DOPE & Click chemistry	Gamma-proteobacteria	gamma 1 symbiont of *O. algarvensis*	35%	Yellow
EUB338	GCT GCC TCC CGT AGG AGT	16S rRNA	1x 6-FAM	Click chemistry	Bacteria	Most Bacteria	35%	Green
EUB338	GCT GCC TCC CGT AGG AGT	16S rRNA	2x 6-FAM	Click chemistry	Bacteria	Most Bacteria	35%	Green
EUB338	GCT GCC TCC CGT AGG AGT	16S rRNA	3x 6-FAM	Click chemistry	Bacteria	Most Bacteria	35%	Green
EUB338	GCT GCC TCC CGT AGG AGT	16S rRNA	4x 6-FAM	Click chemistry	Bacteria	Most Bacteria	35%	Green
EUB338	GCT GCC TCC CGT AGG AGT	16S rRNA	4x Atto488	Click chemistry	Bacteria	Most Bacteria	35%	Green
NON338	ACT CCT ACG GGA GGC AGC	16S rRNA	4x 6-FAM	Click chemistry	Non-sense	none	n/a	Green
NON338	ACT CCT ACG GGA GGC AGC	16S rRNA	4x Atto488	Click chemistry	Non-sense	none	n/a	Green
DSS658	TCC ACT TCC CTC TCC CAT	16S rRNA	Alexa 594 Tyramide	HRP - label	Delta-proteobacteria	delta 1 & 4 symbiont of *O. algarvensis*	60%	Red
Gam42a	GCC TTC CCA CAT CGT TT	23S rRNA	Alexa 488 Tyramide	HRP - label	Gamma-proteobacteria	gamma 1 & 3 symbiont of *O. algarvensis*	35%	Green

SI Table 1: Oligonucleotide probes used in this study. Probe name, nucleotide sequence – modified nucleotide-fluorochrome complexes are indicated by an underscore for MiL-FISH probes, target gene, label type, label synthesis, target taxon or higher, target species and probe colour during imaging

Label	EUB338 Probe signal	NON338 probe signal	S/N ratio
1x EUB338	60.8	14.0	4.3
2x EUB338	105.6	8.0	13.2
3x EUB338	141.3	6.0	23.6
4x EUB338	173.3	5.0	34.7
LR-White mono-FISH, 3h	21.3	4.6	4.6
LR-White mono-FISH, 19h	69.3	14.9	4.7
LR-White 4x labelled MiL-FISH, 3h	70.9	10.0	7.1

SI Table 2: Signal to noise ratio (S/N) for 1x, 2x, 3x and 4x 6-FAM labelled EUB338 probe to the 4x labelled nonsense probe NON338 with mono-FISH hybridisation buffer on *Gramella forsetii*. For imaging with 4x labelled probes lower exposure times resulted in higher S/N ratio. LR-White sections of *O. algarvensis* targeting bacterial symbionts with EUB338 for hybridisation times of both 3 and 19 hours resulted in lower S/N ratio than MiL-FISH on LR-White sections.

SI Figure 1: Melting curve in grey scale (a/u) of 1x, 2x and 4x times 6-FAM labelled CF319a on *Gramella forsetii* hybridised under increasing denaturing conditions with standard FISH buffer in triplicate. Signals for all label types decrease between 30 and 40 % formamide as previously described for CF319a.

SI Figure 2: Three optimisation approaches for CLASI-FISH using MiL-FISH probes. A1.) CLASI-FISH with a repertoire of four probes targeting four sites on the 16S rRNA labelled with various fluorochromes A, B, C & D. A2.) MiL-FISH probe targeting only one site on the 16S rRNA labelled with the same repertoire of fluorochromes A, B, C, & D. B) Two MiL-FISH probes targeting two sites on the 16S rRNA carrying the same label combination for an increase in probe-conferred signal. C) Two MiL-FISH probes carrying a repertoire of eight fluorochromes, A-H, for an increase of label combinations.

SI Figure 3: Schematic of multi-labelled oligonucleotide probes and target organisms for the hybridisation of seven marine microbial groups. The RGB colours red, blue and green are combined by mixing of fluorochrome dye moieties on probes to create magenta, cyan, yellow and white.

References

1. **Schlesner H**. 1986. Pirella marina sp. nov., a budding, peptidoglycan-less bacterium from brackish water. Syst Appl Microbiol **8**:177–180.

2. **Schwedt A**, **Kreutzmann AC**, **Polerecky L**. 2011. Sulfur respiration in a marine chemolithoautotrophic Beggiatoa strain. Front Microbiol **2**:276.

3. **Huber G**, **Spinnler C**, **Gambacorta A**, **Stetter KO**. 1989. Metallosphaera sedula gen, and sp. nov. Represents a New Genus of Aerobic, Metal-Mobilizing, Thermoacidophilic Archaebacteria. Syst Appl Microbiol **12**:38–47.

4. **Behrens S**, **Ruhland C**, **Inacio J**, **Huber H**, **Fonseca A**, **Spencer-Martins I**, **Fuchs BM**, **Amann R**. 2003. In Situ Accessibility of Small-Subunit rRNA of Members of the Domains Bacteria, Archaea, and Eucarya to Cy3-Labeled Oligonucleotide Probes. Appl Environ Microbiol **69**:1748–1758.

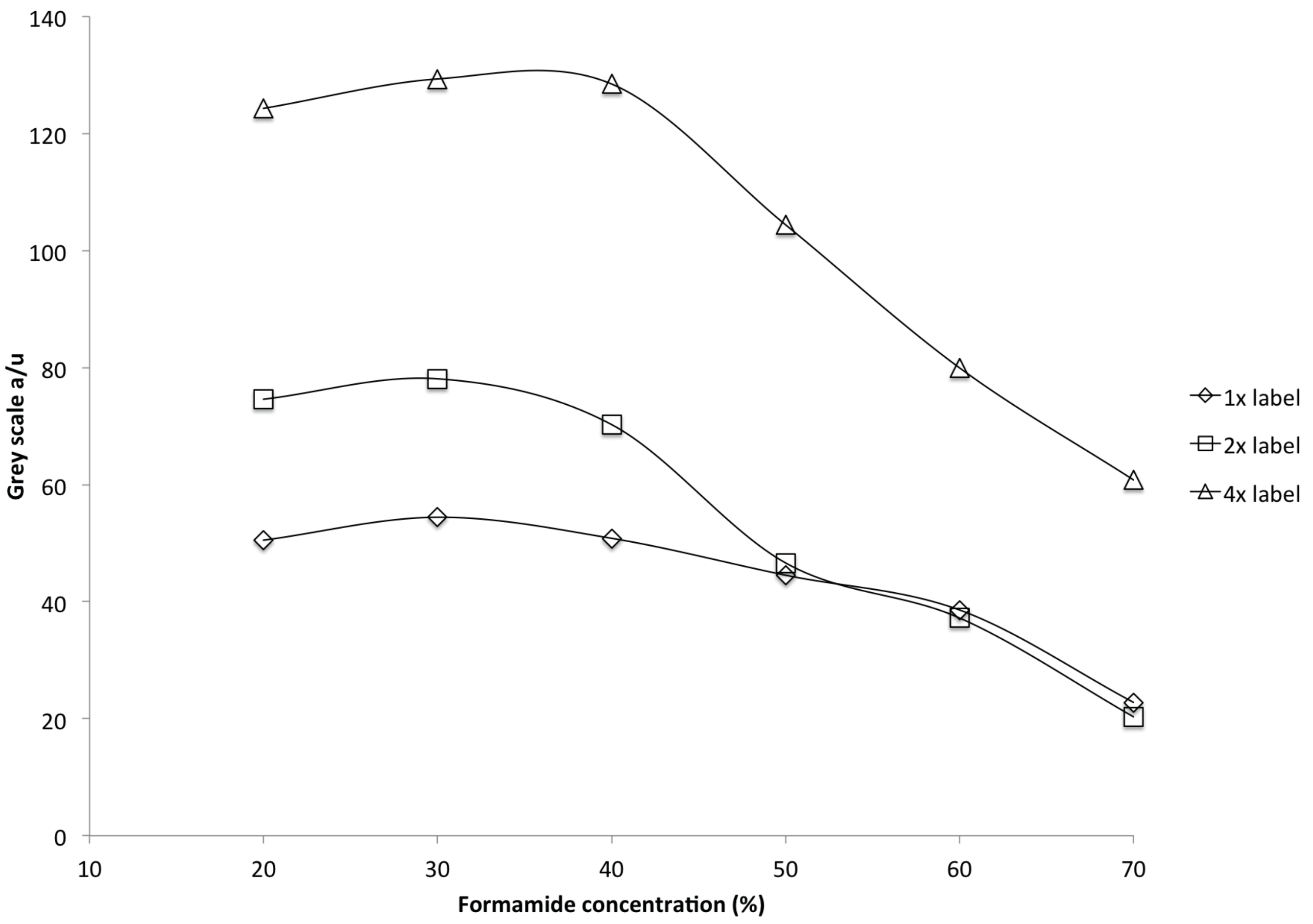

SI Figure 1: Melting curve in grey scale (a/u) of 1x, 2x and 4x times 6-FAM labelled CF319a on *Gramella forsetii* hybridised under increasing denaturing conditions with standard FISH buffer in triplicate. Signals for all label types decrease between 30 and 40 % formamide as previously described for CF319a.

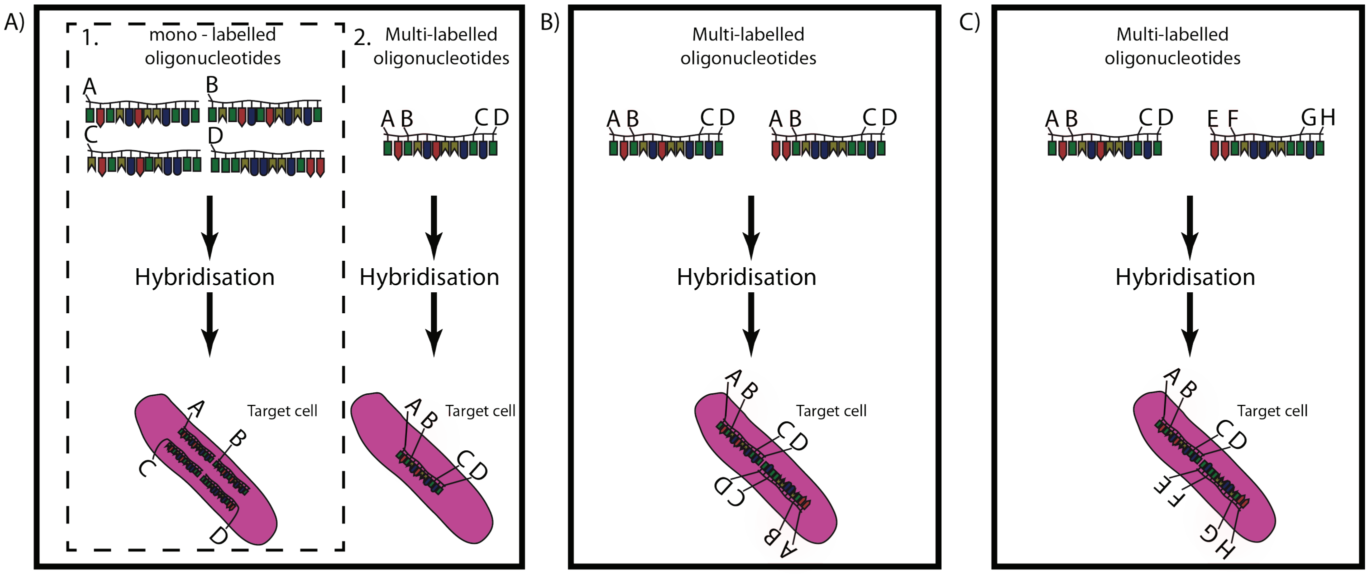

SI Figure 2: Three optimisation approaches for CLASI-FISH using MiL-FISH probes. A1.) CLASI-FISH with a repertoire of four probes targeting four sites on the 16S rRNA labelled with various fluorochromes A, B, C & D. A2.) MiL-FISH probe targeting only one site on the 16S rRNA labelled with the same repertoire of fluorochromes A, B, C, & D. B) Two MiL-FISH probes targeting two sites on the 16S rRNA carrying the same label combination for an increase in probe-conferred signal. C) Two MiL-FISH probes carrying a repertoire of eight fluorochromes, A-H, for an increase of label combinations.

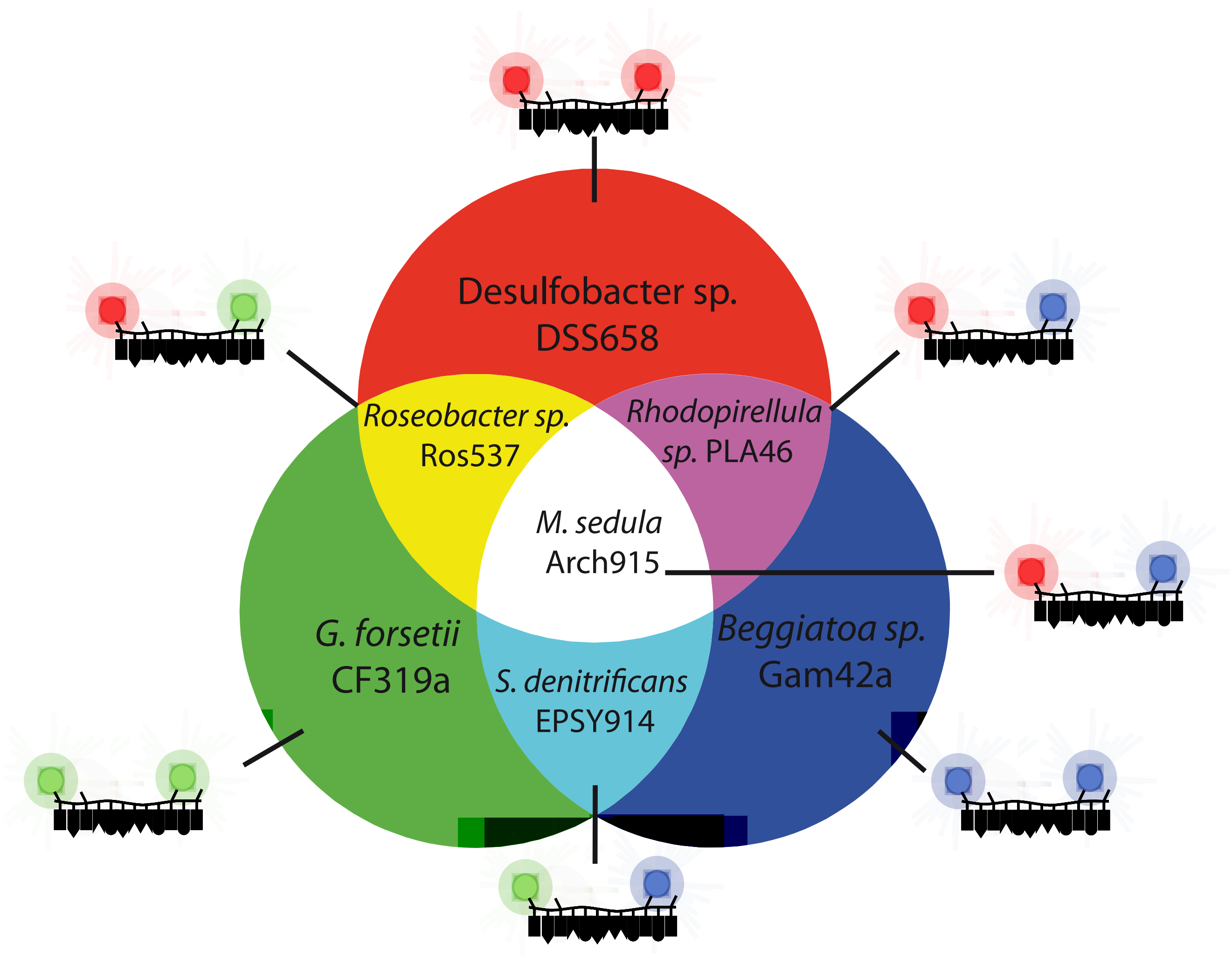

SI Figure 3: Schematic of multi-labelled oligonucleotide probes and target organisms for the hybridisation of seven marine microbial groups. The RGB colours red, blue and green are combined by mixing of fluorochrome dye moieties on probes to create magenta, cyan, yellow and white.
